# High Risk Blueberry Viruses by Region in North America; Implications for Certification, Nurseries, and Fruit Production

**DOI:** 10.3390/v10070342

**Published:** 2018-06-26

**Authors:** Robert R. Martin, Ioannis E. Tzanetakis

**Affiliations:** 1 United States Department of Agriculture–Agricultural Research Service, Horticultural Crops Research Unit, Corvallis, OR 97331, USA; 2 Department of Plant Pathology and Cell and Molecular Biology Program, Division of Agriculture, University of Arkansas System, Fayetteville, AR 72701, USA; itzaneta@uark.edu

**Keywords:** blueberry fruit drop associated virus, blueberry latent virus, blueberry leaf mottle virus, blueberry mosaic associated virus, blueberry red ringspot virus, blueberry scorch virus, blueberry shock virus, blueberry shoestring virus, blueberry virus A, peach rosette mosaic virus, tobacco ringspot virus, tomato ringspot virus, detection, blueberry certification

## Abstract

There is limited information on the distribution of blueberry viruses in the U.S. or around the world other than where the viruses were first discovered and characterized. A survey for blueberry viruses was carried out in the U.S. in 2015–2017. Most blueberry viruses have been characterized to the point that sensitive diagnostic assays have been developed. These assays are based on ELISA or variations of PCR, which were employed here to determine the presence of blueberry viruses in major blueberry production and nursery areas of the U.S. The viruses included in this study were: blueberry fruit drop (BFDaV), blueberry latent (BlLV), blueberry leaf mottle (BLMoV), blueberry mosaic (BlMaV), blueberry red ringspot (BRRV), blueberry scorch (BlScV), blueberry shock (BlShV), blueberry shoestring (BlSSV), blueberry virus A (BVA), peach rosette mosaic (PRMV), tobacco ringspot (TRSV), and tomato ringspot (ToRSV). In the Pacific Northwest BlShV was the most widespread virus, with BlScV and ToRSV detected in a limited number of fields in Oregon and Washington, but BlScV was widespread in British Columbia. In the upper midwest, the nematode-borne (ToRSV, TRSV), aphid-transmitted (BlSSV and BVA) and pollen-borne (BLMoV) viruses were most widespread. In the northeast, TRSV, ToRSV, and BlScV, were detected most frequently. In the southeast, BRRV and BNRBV were the most widespread viruses. BlLV, a cryptic virus with no known symptoms or effect on plant growth or yield was present in all regions. There are other viruses present at low levels in each of the areas, but with the lower incidence they pose minimal threat to nursery systems or fruit production. These results indicate that there are hotspots for individual virus groups that normally coincide with the presence of the vectors. The information presented highlights the high risk viruses for nursery and fruit production each pose a different challenge for control.

## 1. Introduction

In the near past blueberry production was primarily concentrated in North America, with over 80% of world production in 2003 [1]. There were major expansions in production in North America in the 1990s and 2000s [2] but also worldwide; such that North America production accounted for less than 50% of world production in 2016 [3,4]. Approximately 655,000 metric tons of highbush blueberries were produced worldwide in 2016, a 16% increase from two years prior [4]. In the U.S. there was a 70% and 106% increase in acreage and production, respectively, from 2007 to 2016. Additionally, there were 206,500 metric tons of lowbush (wild) blueberries harvested in 2016 [4,5], which were produced primarily in Maine and eastern Canada. With the rapid increase in blueberry production worldwide, there is pressure on the nursery systems to increase production and plants are often being sourced from nurseries in other states, countries or continents resulting in increased international movement of planting material.

The movement of large volumes of vegetative plant material has inherent risks in terms of biosecurity [6,7]. Symptoms by many viruses vary by cultivar making visual inspections inadequate to assess plant health status. The 2016 APS *Blueberry, Cranberry, and Lingonberry Compendium* lists 13 viruses known to infect highbush blueberry [8]. Blueberry latent spherical virus has only been reported from Japan and was not included in the survey. Some of the viruses such as blueberry scorch virus (BlScV, [9]) can cause severe symptoms in some cultivars, whereas it remains symptomless or induce very mild symptoms in others. Blueberry mosaic associated virus (BlMaV, [10]) can be symptomless or develop very striking symptoms of yellow, pink and green mosaic on different branches of the same bush or an infected plant of the same cultivar may remain symptomless entirely. In the case of Blueberry virus A (BVA, [11]), the virus is symptomless in single infections but may be involved in a disease complex in Michigan [12,13] where plants infected with BVA exhibit a bronze leaf curl symptom.

There is an effort in the U.S. to harmonize blueberry certification programs (for more information visit ncpnberries.org), which has been the impetus for this study. For effective certification programs, understanding the viruses that pose the greatest risk for reinfection in nurseries can be used to develop best management practices. The objectives of this work were to identify the high risk blueberry viruses by production region in the North America and make this information available to regulators, and nursery managers. The outcome of such actions will be improved certification systems with higher quality plants that benefit fruit producers and consumers alike.

## 2. Materials and Methods

### 2.1. Plant Material

Samples were collected from the field randomly. They were brought back or shipped to the USDA-ARS Horticultural Crops Research Laboratory in Corvallis, Ore. or the Department of Plant Pathology at the University of Arkansas between 2015 and 2017. Areas of collection were the Pacific Northwest (Oregon and Washington in the U.S. and British Columbia, Canada), the Midwest (Michigan, Wisconsin), the Southeast (Arkansas, Florida, Georgia, and North Carolina), and the Northeast (New Jersey, New York, and Pennsylvania). All samples consisted of the youngest but fully expanded leaves

### 2.2. Nucleic Acid Extractions

Total nucleic acids (TNA) were extracted from leaf samples using the buffers described previously [14,15]. Tissue was frozen with liquid nitrogen and then homogenized using a SPEX SamplePrep 2010 Geno/Grinder (Thomas Scientific, Swedesboro, NJ, USA) following the manufacturer’s recommended procedure. Once the tissue was powdered, 400 μL of extraction buffer [16] and 200 μL potassium acetate (3.8 M K, 5.8 M acetate) were added to each tube and mixed using the Geno/Grinder at 1200 rpm for 1 min. The plates were then centrifuged at 4000 rpm for 15 min and the supernatant used for total nucleic acid extraction using a MagMax Express 96 robot (Applied BioSystems, Foster City, CA, USA) according to manufacturer’s recommendations. Note that after the clarification step, all buffers were as recommended by the manufacturer. The purified nucleic acids were used immediately for downstream reactions or stored at −80 °C until use.

### 2.3. Detection by ELISA

For ELISA, leaf samples were homogenized using a Pollähne roller press with buffer added to the rollers using a peristaltic pump. The grinding buffer used was that described in Martin and Bristow [17]. For BLMoV, PRMV, and TRSV, the ELISA testing was done using commercial kits (AGDIA Inc., Elkhart, IN, USA) according to the manufacturer’s recommendations with the exception of the grinding buffer, which was as described previously [17]. For BlScV, BlShV, and ToRSV, antibodies produced in-house and for BlSSV antibodies obtained from A.C. Schilder (Michigan State University, East Lansing, MI, USA) were used. A_405_ values were determined using the ELx808 plate reader (BioTek U.S., Winooski, VT, USA). Samples were considered positive if the absorbance values were three times or greater than that obtained in wells loaded with buffer rather than leaf sap.

### 2.4. Detection by PCR and RT-PCR

Blueberry fruit drop associated virus (BFDaV), blueberry latent virus (BlLV), blueberry mosaic associated virus (BlMaV), blueberry necrotic ring blotch virus (BNRBV), blueberry red ringspot virus (BRRV), blueberry virus A (BVA), and as well as an internal control standard [15] were tested by RT-PCR whereas blueberry red ringspot virus was detected by PCR. The primer pairs, annealing temperatures, and expected amplicon sizes for the PCR reactions are listed in Table 1.

Reverse transcription reactions (50 μL) consisted of 50 mM Tris acetate (pH 8.4), 75 mM potassium acetate, 8 mM magnesium acetate, 20 mM DTT, 0.4 mM dNTPs, 0.3 μg random primers, and 60 U Maxima reverse transcriptase (Thermo Fisher Scientific, Waltham, MA, USA). The solution was vortexed, centrifuged briefly, incubated at room temperature for 2 min, and then 50 °C for 60 min. The reactions were terminated by 5 min incubation at 85 °C. The cDNA constituted 10% of the total PCR volume. Before virus detection, all samples were evaluated for quality of the RNA by carrying out amplification of the *NADH* dehydrogenase ND-2 subunit as described [15]. Virus-specific PCR reactions (Table 1) were carried out using a program consisting of an initial denaturation for 5 min at 94 °C followed by 40 cycles with denaturation for 30 s at 94 °C, annealing for 30 s at 53–58 °C depending on the primers (Table 1) and extension for 60 s at 72 °C, with a final 10 min extension step at 72 °C. Green Taq DNA polymerase (GenScript, Piscataway Township, NJ, USA) was used according to the manufacturer’s recommendation for all PCR reactions. Amplification products were resolved by electrophoresis through a 2% agarose gel containing ethidium bromide. Representative amplicons for each virus were sequenced to confirm their identity. In the case of BFDaV, all amplicons were sequenced since it was only reported in one county in northwest Washington and in British Columbia, Canada.

## 3. Results and Discussion

**Figure 1:**
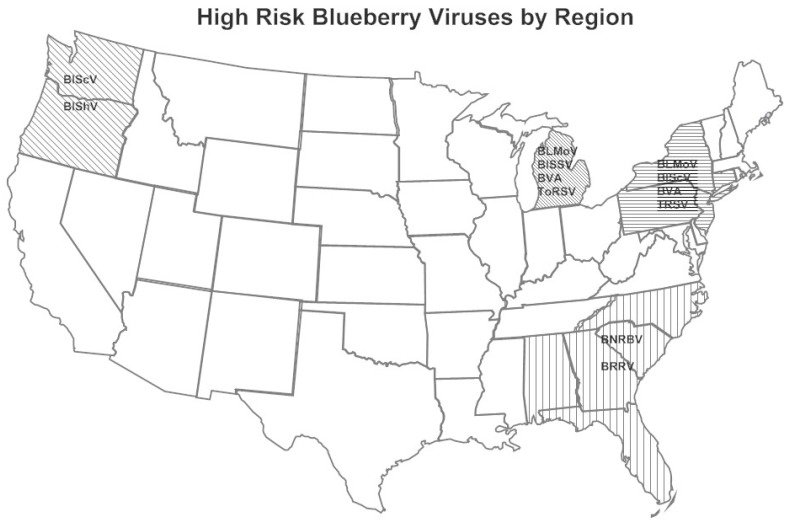
High-risk blueberry viruses by production area in the United States. Blueberry leaf mottle virus (BLMoV), blueberry necrotic ring blotch virus (BNRBV), blueberry red ringspot virus (BRRV), blueberry scorch virus (BlScV), blueberry shock virus (BlShV), blueberry shoestring virus (BlSSV), blueberry virus A (BVA), tobacco ringspot virus (TRSV), and tomato ringspot virus (ToRSV). Minor viruses by production area are listed in Table 2.

The viruses detected in blueberry in the major production areas in the U.S. are shown in Table 2 and the highest risk viruses are shown in Figure 1. BlLV was present in all regions surveyed yet is of little concern since it is a symptomless virus in single infections and it does not have adverse effects when co-infecting with other viruses in mixed infections [18]. BlMaV, an ophiovirus [23], was also present in all locations, generally at low incidence. BLMaV causes little damage as it only affects one or a few branches on a bush [24] with the exception of the cultivar “Brigitta”. The virus was transmitted by *Olpidium virulentus* [16] very slowly, but only in some fields.

The majority of the samples obtained from California were from nurseries, yet the BlShV and BlMaV positive samples were from production fields or breeding plots.

In the Pacific Northwest, most samples were collected in Oregon and Washington with the exception of 184 samples collected in British Columbia, Canada primarily because BFDaV was observed in two fields there [19]. BFDaV was very limited in distribution and only found in the cultivar “Bluecrop” in the Fraser River Valley, in southwest British Columbia and northwest Washington. However, there was a single, symptomless (no fruit drop) BFDaV-infected plant of the cultivar “Aron” at the National Clonal Germplasm Repository in Corvallis, Oregon. The plant originated in Finland and was brought to the repository in the 1980s. Still none of the surrounding plants in the field or screenhouse plantings were positive for this virus. BlShV and BlScV were the two major viruses detected in the Pacific Northwest. BlShV is very widespread occurring in more than 40% of the field samples collected. BlShV occurs throughout this region whereas BlScV is much more limited in distribution, but still a concern for the industry, since it is aphid-transmitted and can cause a near 100% crop loss in some cultivars [17].

In the southeast U.S.A., there were two viruses that were common, BRRV and BNRBV with the latter detected only in this region. BRRV has been reported sporadically in other regions but it is considered an important pathogen in New Jersey where it was first identified [25]. The absence of TRSV was unexpected since it is widely distributed in blackberries especially in North Carolina [26]. It may be that because blueberries are grown in a low pH, high organic matter soil [27] that makes it not conducive to the nematode vector.

In the northeast BLMoV, BlScV, BVA, and TRSV were the most common viruses. BlScV is aphid-transmitted as is probably the case of BVA, a closterovirus [11]. TRSV was quite common with the other two nematode-transmitted viruses, PRMV and ToRSV present, though at lower levels. BLMoV, although is taxonomically related to the three aforementioned viruses has no documented nematode vector, but is pollen-borne in highbush blueberry [28].

In the upper Midwest, the aphid or hypothesized aphid-transmitted BlSSV and BVA were the most common viruses suggesting that there may be high aphid populations in this region, or an efficient aphid vector of these two viruses. All three nematode transmitted viruses, PRMV, ToRSV, and TRSV, were also present in this region.

There is little information on virus resistance in blueberries. “Jersey” is reported to be immune to BRRV [25]. “Bluecrop” has a high level of field resistance to ToRSV even though it is not immune to the virus [8]. In the case of BlShV, a pollen-borne virus, there are significant differences between cultivars on the rate of virus spread through a field [29]. In a cultivar trial in Oregon with high BlShV pressure some cultivars became infected in 1–3 years (“Bluegold”, “Nui”, “Ozarkblue”, “Hardyblue”), whereas for others (such as “Bluecrop”, “Legacy”, and “Toro”) it took more than 15 years before the first infection [29]. 

The information from this survey highlights the most prevalent viruses in blueberry by production region in the North America. Several viruses were very limited in distribution, the extreme example being BFDaV which was detected in only one field in northwest Washington State and in several fields in adjoining British Columbia, Canada. In the U.S. efforts are underway to eradicate this virus. It was only detected in the cultivar “Bluecrop” in the field, but not in over 300 samples tested from other cultivars planted adjacent to fields with the infected material. The presence of the virus in “Aron” at the National Clonal Germplasm Repository in Corvallis, Oregon is of concern but no other cultivar in the block tested positive for the virus. BNRBV was only detected in the southeast U.S.A. where it was quite common. BRRV was primarily a problem in the southeast U.S.A., though there was a single positive from the northeast U.S., where it was originally described [25]. BRRV has been reported in Michigan, though it does not appear to spread [30].

BlShV was widespread in the Pacific Northwest, with over 40% of the samples testing positive for this virus. This was clearly the most widespread of any of the viruses within a production region. This virus is pollen-borne; plants generally lose one full year’s crop but then recovers to full production [31]. For this reason, growers tend to leave recovered plants in the field rather than replanting. BlScV was detected primarily in the Pacific Northwest and the northeast U.S., where it has been reported previously.

The information on high risk viruses for blueberries in the U.S. and British Columbia, Canada can be used by growers to manage virus diseases in production fields, by State Departments of Agriculture to manage more efficiently certification programs and by nurseries to inform the development of their pest management plan. Certification programs are based on best management practices (BMPs) to minimize risk of pathogen infection, combined with testing to ensure effectiveness [32]. Testing should focus on pathogens most likely to spread in the area where a nursery is located. Few if any of the viruses in blueberry cause visual symptoms in all cultivars, which highlights the importance of performing nursery stock testing and not rely on visual inspections. In nurseries, the goal should be to control all viruses to minimize the risk of distributing symptomless viruses to production fields. This is especially important with the movement of plant materials across state and country borders, where introducing new viruses to a region could risk production. If nurseries obtain material from a trusted source (e.g., clean plant center) where the material has been tested and found free of known viruses, then monitoring should focus on viruses spread most rapidly in the area, with some testing for other viruses at the top two tiers of propagation [6].

Nematodes are one of the more challenging vectors to manage in a nursery system since fumigation is only effective in the upper layers of soil and nematodes below the depth of treatment escape and can repopulate the site. Many blueberry nurseries are in canyards, potted plants maintained on a bed of course gravel >5 cm thick, or on plastic laid over the soil. Also, the blueberries are grown in soilless media. This growing system avoids the risk of nematode transmitted viruses. From a total of 79 samples no parasitic nematodes were detected in either the potting media or below the plastic barrier [33]. This approach is the better alternative to fumigation for managing nematode transmitted viruses in nurseries.

For some commodities such as potato, where the growers only have the crop in the field for one year, there tolerances for virus incidence built into certification schemes. With long term crops such as blueberry, even a very low level of infection is problematic for fruit producers since it takes 5–8 years for plant to come into full production. No certification system will be 100% effective, even if every plant was fully tested prior to planting. Thus, it is important for producers to monitor their fields for infection and rogue infected plants promptly to increase the productive life of a field. With good certification programs, there should be few plants that will need to be removed, but if we look at plant production as a system, the final check is to monitor what growers planted in the production fields.

## Figures and Tables

**Table 1:** Primer pairs, annealing temp, and amplicon size for each of the viruses tested by PCR

Virus	Primer Pair	Annealing Temp	Amplicon Size (Ref) ^1^
BlLV	F: CTTATCAGAGCTTCTTCAGACTGG R: TCGTCACCCGCACATTTC	55	391 [18]
BFDaV	F: GACAACAGCATCTACATCTCTGC R: GGTCGTTCTACCACGTTTCTG	53	395 [19]
BlMaV	F: CCWGTATCAAGCATAGTYACAAG R: AAGAAGGTRGTGATTGAGA	58	254 [20]
BNRBV	F: CCAGTTTGGAGGAATTGCAT R: GCGTTTCAGCACCACTAAC	55	432 [21]
BRRV	F: ATCAGTCCCAGAAGAAAAGAAGTA R: TCCGAAAAATAGATAGTGTCAGC	56	548 [22]
BVA	F: AACTCATGGTTAAGCGTGAG R: AGTCCTGAGACTTATCGAAC	55	270 [12]
*NADH (β)*	AGTAGATGCTATCACACATACAAT GGACTCCTGACGTATACGAAGGATC (note DNA has intron and yields ~1100 bp band)	55	721 [15]

^1^ (Ref) Citation with the primer information, PCR conditions and amplicons size.

**Table 2:** Viruses detected in major highbush blueberry production areas in North America

Acronym ^1^	Genus	Transmission	Laboratory Detection	California	Regional Occurrence				Positives ^#^
					Northwest	Southeast	Northeast	Midwest	
BFDaV	?	?	RT-PCR???	0/148	64/1244 *	0/221	0/555	0/411	64
BlLV	*Amalgavirus*	pollen/seed ◊	RT-PCR	5/148	22/1244	15/221	48/555	2/411	92
BLMoV	*Nepovirus*	nematodes? □◊	ELISA	0/148	0/1244	0/221	**18/555**	**12/411**	30
BlMaV	*Ophiovirus*	Olpidium/?	RT-PCR	9/148	18/1244	2/221	20/555	35/411	84
BNRBV	*Blunervirus*	eriophyid mites?	RT-PCR	0/148	0/1244	**26/221**	0/555	0/411	26
BRRV	*Soymovirus*	?	PCR	0/148	0/1244	**46/221**	1/555	0/411	47
BlScV	*Carlavirus*	aphids/non-persistent	ELISA/RT-PCR	0/148	**43/1244**	0/221	**70/555**	1/411	114
BlShV	*Ilarvirus*	pollen/seed ◊	ELISA/RT-PCR	8/148	**558/1244**	0/221	0/555	0/411	566
BlSSV	*Sobemovirus*	aphids/non-persistent	ELISA	0/148	0/1244	0/221	4/555	**49/411**	53
BVA	*Closterovirus*	aphids/Semi-persistent?	RT-PCR	0/148	0/1244	0/221	**29/555**	**90/411**	119
PRMV	*Nepovirus*	nematodes/persistent □◊	ELISA	0/148	0/1244	0/221	2/555	6/411	8
TRSV	*Nepovirus*	nematodes/persistent □◊	ELISA	0/148	0/1244	0/221	**42/555**	4/411	46
ToRSV	*Nepovirus*	nematodes/persistent □◊	ELISA	0/148	5/1244	0/221	6/555	13/411	24

^1^ BFDaV = Blueberry fruit drop associated virus, BlLV = Blueberry latent virus, BLMoV = Blueberry leaf mottle virus, BlMaV = Blueberry mosaic associated virus, BNRBV = Blueberry necrotic ring blotch virus, BRRV = Blueberry red ringspot virus, BlScV = Blueberry scorch virus, BlShV = Blueberry shock virus, BlSSV = Blueberry shoestring virus, BVA = Blueberry virus A, PRMV = Peach rosette mosaic virus, TRSV = Tobacco ringspot virus, ToRSV = Tomato ringspot virus; * All BFDaV positives were from 3 fields in the Fraser River Valley in Whatcom county Washington and British Columbia, Canada; with the exception of 1 sample from the National Clonal Germplasm. Repository in Corvallis, OR, where a single plant of cultivar “Aron” tested positive for BFDaV. ◊ Also transmitted by pollen feeding arthropods. □ Pollen and seed transmitted. ^#^ Total number of positives for virus in the survey. Bold font highlights highest risk viruses in the production area.
